# A case of gastric cardia telangiectasias and primary biliary cirrhosis with hemorrhage

**DOI:** 10.1002/ccr3.3147

**Published:** 2020-07-17

**Authors:** Jinsheng Guo

**Affiliations:** ^1^ Department of Gastroenterology and Hepatology Zhongshan Hospital Fudan University Shanghai Institute of Liver Diseases Shanghai China

**Keywords:** anemia, gastric cardia telangiectasias, portal hypertension, primary biliary cirrhosis

## Abstract

In patients presenting with persistent anemia and gastric cardia telangiectasias, a potential etiology of portal hypertension due to chronic liver disease, especially primary biliary cirrhosis, should be considered. Drugs for treating specific liver disease and lowering portal hypertension are effective strategies to prevent hemorrhage.

## Graphical abstract

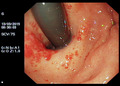

## 1 INTRODUCTION

Endoscopic cauterization of visible telangiectasias may be considered in the patients with gastrointestinal telangiectasias and anemia not responding to iron supplementation.^1^, ^2^, ^3^ However, acquired diseases such as portal hypertension caused by chronic liver diseases must be excluded and appropriately treated with drugs for specific liver disease and reducing portal hypertension. Here, we present a case of a male patient with severe anemia due to gastric cardia telangiectasias. His case was complicated with primary biliary cirrhosis (PBC) after screening for potential liver diseases. Chronic liver disease may cause portal hypertension which is a contraindication to endoscopic cauterization management.

## 2 CASE PRESENTATION

The case report was approved by the ethics committee of Zhongshan Hospital affiliated to Fudan University.

A 67‐year‐old man presented to the hospital with severe anemia. He had been diagnosed with cardia telangiectasias by gastroendoscopy and treated with blood transfusions, proton pump inhibitors, iron supplement, hemocoagulase, octreotide, thalidomide during the past 5 years by several hospitals. However, the symptoms of dizziness and fatigue occurred repeatedly with blood tests showing severe iron‐deficient anemia and hemoglobin levels less than 60 g/L. He had melena intermittently, insomnia, but no symptoms of epistaxis, hematemesis, dysphagia, arthralgia, and Raynaud’s phenomenon. He had no history of hypertension, diabetes, and coronary heart disease, and no history of any nonsteroidal anti‐inflammatory drug (NSAID) use. He had a previous history of dysentery at teenager but no hepatitis and tuberculosis, and other infectious diseases. He denied smoking and drinking habits. He also denied a family history of genetic diseases and infectious diseases. He worked as a facility administrator and retired seven years before the presentation and had no history of chemical exposure.

At presentation, his vitals were within normal limits with a body temperature of 36.9°C, pulse rate at 75 beats per minute, and systolic/diastolic blood pressure 122/82 mm Hg. He was noticeably pale and appeared fatigued. He had no signs of jaundice, cutaneous telangiectasias, spider nevi, or Raynaud's phenomenon. Cardiovascular, respiratory, abdominal, and nervous system examinations were unremarkable. Laboratory tests, including blood routine and biochemistry, are shown in Table 1, indicating hypochromic microcytic anemia and mitochondria antibody specific for PBC positive.

**Table 1:** Laboratory data at the presentation

Parameters (abbreviation)	Result	Reference interval	Unit
Blood routine:			
Red blood cell count (RBC)	2.85	4.30 ~ 5.80	10^12^/L
Hemoglobin (Hb)	55	130 ~ 175	g/L
White blood cell count (WBC)	2.31	3.50 ~ 9.50	10^9^/L
Neutrophil	1.4	1.8‐6.3	10^9^/L
Lymphocyte	0.5	1.1‐3.2	10^9^/L
Monocyte	0.34	0.1‐0.6	10^9^/L
Eosinophil	0.02	0.02‐0.52	10^9^/L
Basophil	0.01	0.00‐0.06	10^9^/L
Hematocrit (HCT)	20.7	40‐50	%
Mean red cell volume (MCV)	72.6	82.0‐100.0	fL
Mean corpuscular hemoglobin (MCH)	19.3	27.0 ~ 34.0	pg
Mean corpuscular hemoglobin concentration (MCHC)	266	316‐354	g/L
Platelet (PLT)	102	125‐350	10^9^/L
Stool routine:			
Red cells, mucous, and white blood cells	(−)	(−)	
Lipids	(−)	(−)	
Occult blood	(+)	(−)	
Ova and parasites	(−)	(−)	
Coagulation function:			
Prothrombin time (PT)	13.2	10‐13	Second
International normalized ratio, INR	1.29	0.5‐1.2	
D‐dimer	0.63	0.02‐0.8	mg/L
Liver function tests (LFTs):			
Total bilirubin	7.4	3.4‐20.4	μmol/L
Direct bilirubin	3.0	0.0‐6.8	μmol/L
Total protein	75	65‐85	g/L
Albumin	35	35‐55	g/L
Alanine aminotransferase (ALT)	78	9‐50	U/L
Aspartate aminotransferase (AST)	69	15‐40	U/L
Alkaline phosphatase (AKP）	205	45‐125	U/L
Gamma glutamyltransferase (GGT）	270	10‐60	U/L
Cholinesterase	8212	5000‐12000	U/L
Prealbumin (PA)	0.187	0.25‐0.40	g/L
Renal function tests:			
Urea	8.2	2.9‐8.2	mmol/L
Creatinine	78	44‐115	μmol/L
Uric acid	355	155‐357	μmol/L
Glomerular filtration rate based on CKD‐EPI creatinine equation	89		ml/min/1.73m^2^
Blood glucose	6.2	3.9‐5.6	mmol/L
Blood fat:			
Total cholesterol	3.64	<5.2	mmol/L
Triglyceride	0.76	<1.7	mmol/L
Immunological and inflammatory parameters:			
IgG	18.47	7.00‐16.00	g/L
IgG4	0.44	0.03‐2.00	g/L
IgM	2.03	0.40‐2.30	g/L
C‐reactive protein (CRP)	1.6	0.0‐3.0	g/L
Serum amyloid protein (SAA)	3.1	0.0‐6.4	mg/L
Muscle enzymes:			
Creatine kinase (CK)	35	18.0‐198.0	U/L
Creatine kinase MB isoenzyme	19	0‐23	U/L
Autoantibodies:			
Antinuclear antibody, cytoplasmic pattern	1:1000	(−)	
Smooth muscle (SMA)	(−)	(−)	
Soluble liver antigen/liver pancreas (SLA/LP)	(−)	(−)	
Liver cyrosol (LC1)	(−)	(−)	
Liver kidney microsomal type 1(LKM‐1)	(−)	(−)	
Antimitochondrial antibody（AMA）	(+)	(−)	
Antimitochondrial antibody M2 subtype	(+)	(−)	
Extractable nuclear antigens (ENAs):			
Ribonucleic protein (RNP), Smith (Sm), Ro (single‐strand A, SS‐A), La (single‐strand B, SS‐B), scleroderma‐70 (SCL‐70), JO‐1, PM‐Scl	(−)	(−)	
Proliferating cell nuclear antigen (PCNA)	(−)	(−)	
Anticentromere antibody (ACA)	(−)	(−)	
Antihistone (AHA)	(−)	(−)	
Antinucleosome (AnuA)	(−)	(−)	
Antineutrophil cytoplasmic antibodies (ANCAs):			
Cytoplasmic antineutrophil cytoplasmic antibody (cANCA), perinuclear antineutrophil cytoplasmic antibody (pANCA)	(−)	(−)	
Proteinase 3 (PR3)	<2.00	<20	RU/mL
Myeloperoxidase (MPO)	<2.00	<20	RU/mL
Genetic liver diseases			
Ceruloplasmin (CER)	0.38	0.15‐0.30	g/L
Infectious diseases			
Hepatitis B virus surface antigen (HBsAg)	(−) 01	<1.0	COI
Hepatitis C virus antibody	(−) 0.0402	<1.0	COI
Human immunodeficiency virus antibody	–	–	
Specific antibody of treponema pallidum	–	–	
Tumor markers			
Alpha‐fetoprotein AFP	2.9	<20	ng/mL
Carcinoembryonic antigen (CEA)	1.8	<5	ng/mL
Carbohydrate antigen 19‐9 (Ca19‐9)	7.6	<34	U/mL

Abbreviations: (−), negative result; (+): positive result.

**Figure 1:**
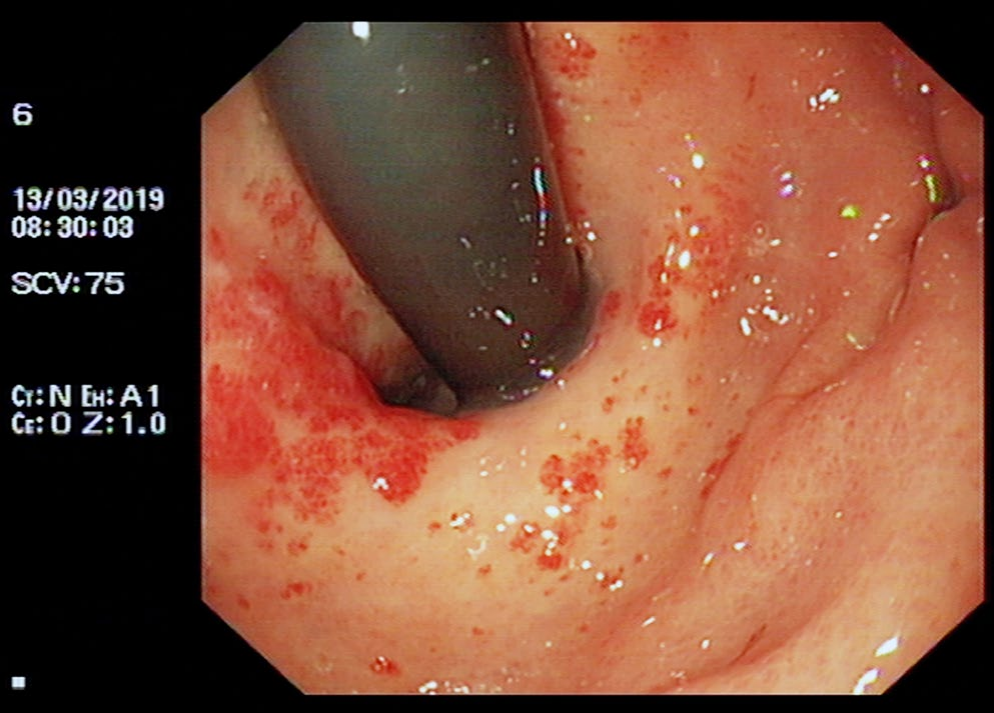
Gastroendoscopy shows cardia telangiectasias, with significant dilation of gastric capillary extending from the cardia to the fundus of stomach, partial fusion

Telangiectasias at the cardia of the stomach were found under gastroscope (Figure 1). Esophageal mucosa was normal. Endoscopic therapy using argon plasma coagulation (APC) was first considered for treating the patients, but the operation was canceled by a suspicion that the patient may suffer from a potential chronic liver disease.

Previous liver function tests (LFTs) of this patient only recorded mild increases of γ‐glutamyl transferase (GGT). A re‐examination of his LFT showed a normal serum bilirubin level, mild hypoalbuminemia (35g/L), a pattern of cholestatic liver biochemistry noted with increased activities of alanine aminotransferase (ALT) 78U/L, aspartate aminotransferase (AST) 69U/L, alkaline phosphatase (ALP) 205U/L, and GGT 270 U/L. He also had a mild prolonged prothrombin time (13.2 seconds) and a lower platelet count (102 × 10^9/L). Hepatitis B surface antigen (HBsAg) and hepatitis C antibody (HCV‐Ab) were negative. Positive results were found in testing antinuclear antibody (ANA) (cytoplasmic speckled pattern 1:1000), antimitochondrial antibody (AMA), and antimitochondrial M2 subtype antibody (AMA‐M2). FibroScan showed a liver stiffness of 9.6kPa. Computed tomography angiogram (CTA) of hepatic artery, portal vein, hepatic vein, and inferior vena cava showed mild liver cirrhosis with splenomegaly, without significant abnormality in portal vein system, hepatic vein, and inferior vena cava.

**Figure 2:**
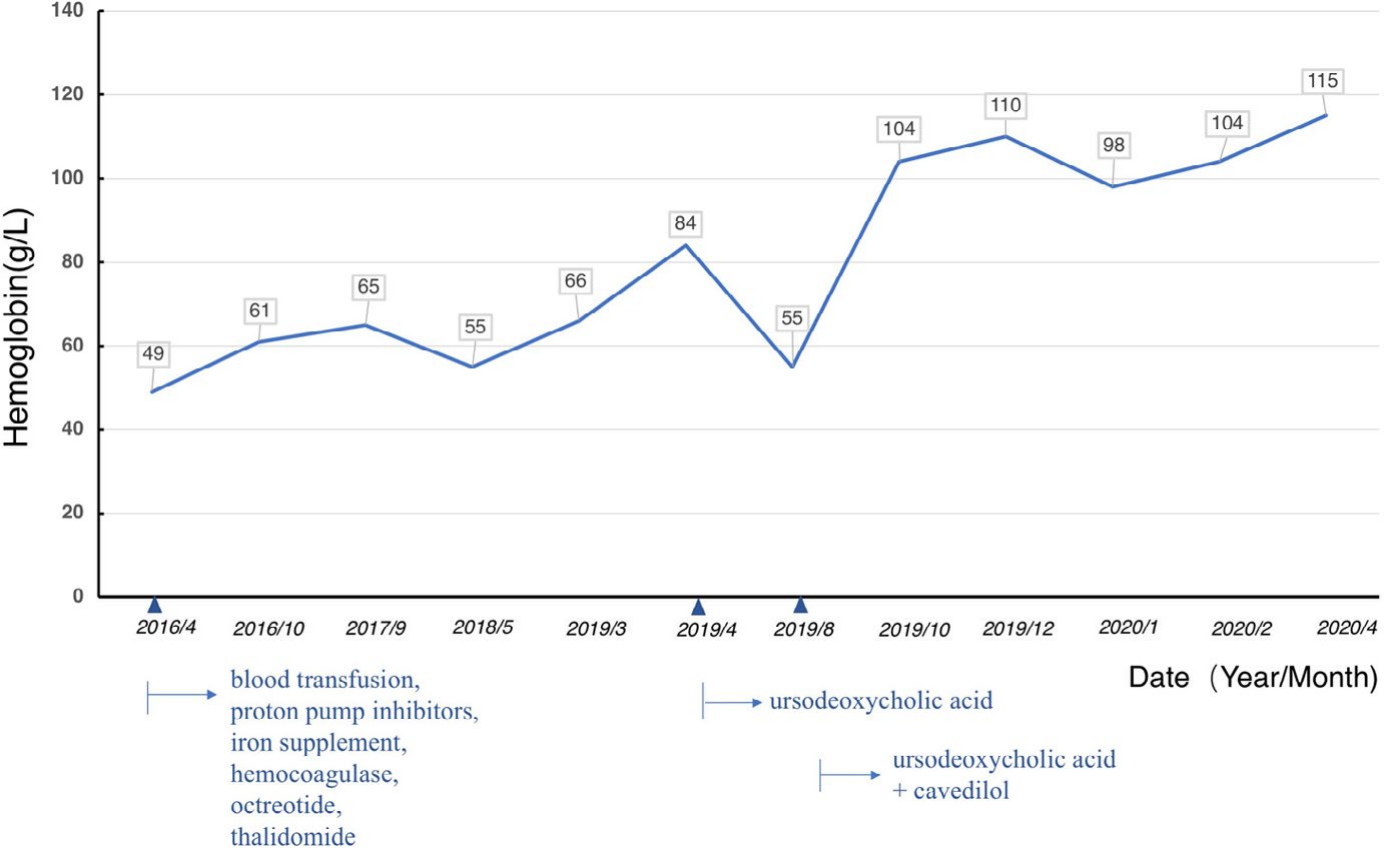
A monitoring of blood hemoglobin levels shows that anemia was corrected when a combination of ursodeoxycholic acid (for treating primary biliary cirrhosis) and carvedilol (for lowering portal vein pressure) was administrated to this male patient

Ursodeoxycholic acid (UDCA, 250 mg PO twice daily) was then prescribed to the patient after a definite diagnosis of primary biliary cirrhosis (PBC) was established. Liver function was improved in months after UDCA treatment, but the amelioration of anemia was only achieved by a further administration of carvedilol (Figure 2). The dosage of carvedilol started with 5 mg once a day as the patient had severe anemia at the beginning. The drug was prescribed as half a tablet (=5 mg) per day first, with blood pressure and pulse rate monitored. The dosage increased to tolerable 5 mg twice daily in months to maintain systolic/diastolic arterial blood pressure around 100 ~ 110/60 ~ 70 mm Hg and resting heart rate 60 ~ 65 beats per minute. The patient had been following up for one year with good compliance. His hematocrit remained stable at 9 ~ 11g/L, and he required no additional blood transfusions.

## 3 DISCUSSION

Telangiectasias as one component of CRST (calcinosis cutis, Raynaud's phenomenon, sclerodactyly, and telangiectasias) and CREST (calcinosis, Raynaud's phenomenon, esophageal dysfunction, sclerodactyly, and telangiectasias) syndromes have been reported to complicate with autoimmune liver disease, especially PBC.^4^, ^5^, ^6^, ^7^, ^8^ The presence of telangiectasias was usually multiple and located at the extremities. The mechanism for the appearance of CRST/CREST in PBC is so far unclear but may be genetically associated with different human histocompatibility leukocyte antigen (HLA) typing.^8^ The patient discussed here presented primarily with refractory anemia and gastric cardia telangiectasias, without other features of CRST/CREST syndrome and other extrahepatic autoimmune conditions such as Sjögren’s syndrome and Hashimoto’s disease.

PBC is characterized by female predominance and disease‐specific serum AMA‐M2 and ANA autoantibodies. PBC may be diagnosed at a later stage in men, potentially reflecting perception bias among clinicians. The incidence of portal hypertension in patients with PBC is significant, however, even in the absence of established cirrhosis, and significant liver function insufficiency. The use of UDCA is recommended as the first‐line, lifelong pharmacotherapy for all patients with PBC by current guidelines.^9^, ^10^ The mechanism of action of UDCA in PBC and other cholestatic disorders includes the following: (a) protection of injured cholangiocytes against the toxic effects of bile acids, (b) stimulation of impaired hepatocellular secretion by posttranscriptional mechanisms including the stimulation of synthesis, targeting and apical membrane insertion of key transporters, and (c) stimulation of ductular alkaline choleresis and inhibition of bile acid–induced hepatocyte and cholangiocyte apoptosis.^11^ Carvedilol is a nonselective beta‐blocker (NSBB) that is more powerful in reducing portal vein pressure than traditional NSBBs because, in addition to β‐blockade, it relaxes the increased hepatic vascular tone due to anti‐α‐adrenergic activity. In addition, carvedilol has its maximal effects on portal hypertension already at low dosages. It is, therefore, becoming the most widely used beta‐blocker for the management of portal hypertension in compensated cirrhosis.^12^ Persistence anemia with cardia telangiectasias should consider a potential etiology of portal hypertension due to a chronic liver disease,^1^ especially PBC. Although gastric cardia telangiectasias were not classified as a form of gastroesophageal and gastric varices,^13^ the increased portal pressure of PBC may aggravate telangiectasias in the gastrointestinal tract and aggravate gastrointestinal bleeding. Drugs aiming at treating the specific liver disease and lowering portal hypertension are effective and safe for prophylaxis of the hemorrhage.

## 4 CONCLUSION

Cardia telangiectasias are a rare consequence of portal hypertension, resulting in refractory anemia secondary to gastrointestinal bleeding. Chronic liver diseases, especially primary biliary cirrhosis, should be excluded in patients with an endoscopic finding of cardia telangiectasias, even in males. Drugs targeting specific liver disease and portal hypertension are effective for the prophylaxis of hemorrhage.

## CONFLICT OF INTEREST

None declared.

## AUTHOR CONTRIBUTIONS

JG: took care of the patient in the hospital, collected clinical data and information, and wrote and revised the manuscript.

## INFORMED CONSENT

The patient provided written informed consent for the publication of the details of the diagnosis.

## References

[1] Faughnan ME , Palda VA , Garcia‐Tsao G , et al. International guidelines for the diagnosis and management of hereditary haemorrhagic telangiectasias. J Med Genet. 2011;48(2):73–87. 19553198 10.1136/jmg.2009.069013

[2] Goldman MP , Bennett RG . Treatment of telangiectasia: a review. J Am Acad Dermatol. 1987;17(2 Pt 1):167–182. 3305603 10.1016/s0190-9622(87)70187-x

[3] Checketts SR , Burton PS , Bjorkman DJ , Kadunce DP . Generalized essential telangiectasia in the presence of gastrointestinal bleeding. J Am Acad Dermatol. 1997;37(2 Pt 2):321–325. 9270538

[4] Ito M , Kojima T , Miyata M , et al. Primary Biliary Cirrhosis (PBC)‐CREST (Calcinosis, Raynaud's Phenomenon, Esophageal Dysfunction, Sclerodactyly and Telangiectasia) Overlap Syndrome Complicated by Sjögren's Syndrome and Arthritis. Intern Med. 1995;34(5):451–454. 7647420 10.2169/internalmedicine.34.451

[5] Reynolds TB , Denison EK , Frankl HD , Lieberman FL , Peters RL . Primary biliary cirrhosis with scleroderma, Raynaud's phenomenon and telangiectasia. New syndrome. Am J Med. 1971;50(3):302–312. 5553949 10.1016/0002-9343(71)90218-x

[6] Ishikawa M , Okada J , Shibuya A , Kondo H . CRST syndrome (calcinosis cutis, Raynaud's phenomenon, sclerodactyly, and telangiectasia) associated with autoimmune hepatitis. Intern Med. 1995;34(1):6–9. 7718984 10.2169/internalmedicine.34.6

[7] Shoji I , Takagi T , Kasukawa R . Anti‐centromere antibody and CREST syndrome in patients with primary biliary cirrhosis. Intern Med. 1992;31(12):1348–1355. 1284406 10.2169/internalmedicine.31.1348

[8] Akiyama Y , Tanaka M , Takeishi M , Adachi D , Mimori A , Suzuki T . Clinical, serological and genetic study in patients with CREST syndrome. Intern Med. 2000;39(6):451–456. 10852162 10.2169/internalmedicine.39.451

[9] Lindor KD , Bowlus CL , Boyer J , Levy C , Mayo M . Primary biliary cholangitis: 2018 practice guidance from the American Association for the Study of Liver Diseases. Clin Liver Dis. 2020;15(1):1–2. 10.1002/hep.30145 30070375

[10] Hirschfield GM , Dyson JK , Alexander GJ , et al. The British Society of Gastroenterology/UK‐PBC primary biliary cholangitis treatment and management guidelines. Gut. 2018;67(9):1568–1594. 29593060 10.1136/gutjnl-2017-315259 PMC6109281

[11] European Association for the Study of the Liver . EASL Clinical Practice Guidelines: management of cholestatic liver diseases. J Hepatol. 2009;51(2):237–267. 19501929 10.1016/j.jhep.2009.04.009

[12] Garcia‐Tsao G , Abraldes JG , Berzigotti A , Bosch J . Portal hypertensive bleeding in cirrhosis: risk stratification, diagnosis, and management: 2016 practice guidance by the American association for the study of liver diseases. Hepatology. 2017;65(1):310–335. 27786365 10.1002/hep.28906

[13] Sarin SK . Long‐term follow‐up of gastric variceal sclerotherapy: an eleven‐year experience. Gastrointest Endosc. 1997;46(1):8–14. 9260698 10.1016/s0016-5107(97)70202-5

